# Differential Expression of Circulating Damage-Associated Molecular Patterns in Patients with Coronary Artery Ectasia

**DOI:** 10.3390/biom14010010

**Published:** 2023-12-21

**Authors:** James N. Tsoporis, Andreas S. Triantafyllis, Andreas S. Kalogeropoulos, Shehla Izhar, Angelos G. Rigopoulos, Loukianos S. Rallidis, Eleftherios Sakadakis, Ioannis K. Toumpoulis, Vasileios Salpeas, Howard Leong-Poi, Thomas G. Parker, Ioannis Rizos

**Affiliations:** 1 Keenan Research Centre for Biomedical Science, Li Ka Shing Knowledge Institute, St. Michael’s Hospital, Unity Health Toronto, University of Toronto, 30 Bond St., Toronto, ON M5B 1W8, Canada; shehla.izhar@unityhealth.to (S.I.); howard.leong-poi@unityhealth.to (H.L.-P.); thomas.parker@unityhealth.to (T.G.P.); 2 Second Department of Cardiology, Attikon University Hospital, 12462 Athens, Greece; andreas.triantafyllis@catharinaziekenhuis.nl (A.S.T.); akalogeropoulos@mitera.gr (A.S.K.); arigopoulos@mitera.gr (A.G.R.); lrallidis@med.uoa.gr (L.S.R.); esakadakis@mitera.gr (E.S.); itouboul@med.uoa.gr (I.K.T.); vsalpeas@med.uoa.gr (V.S.); johnrizos@med.uoa.gr (I.R.); 3 Askepeion General Hospital, 16673 Athens, Greece; 4 Hygeia HealthCare Group, Department of Cardiology, Mitera General Hospital, 15123 Athens, Greece

**Keywords:** coronary artery ectasia, inflammation, sRAGE, S100B, S100A12, TLR4, HSP70, DJ-1

## Abstract

Coronary artery ectasia (CAE) is defined as abnormal dilation of a coronary artery with a diameter exceeding that of adjacent normal arterial segment by >1.5 times. CAE is a pathological entity of the coronary arteries and characterized as a variant of coronary atherosclerosis. CAE frequently coexists with coronary artery disease (CAD). While inflammation appears to be involved, the pathophysiology of CAE remains unclear. Damage-associated molecular patterns (DAMPs), defined as endogenous molecules released from stressed or damaged tissue, are deemed as alarm signals by the innate immune system. Inflammatory agents can generate DAMPs and DAMPs can create a pro-inflammatory state. In a prospective cross-sectional study, we enrolled 29 patients with CAE and non-obstructive CAD, 19 patients with obstructive CAD without CAE, and 14 control subjects with normal (control) coronary arteries age- and sex-matched with the CAE patients, to investigate the differential expression of plasma DAMPs. Patients with CAE and non-obstructive CAD had increased plasma levels of the DAMPs S100B, S100A12, HMGB1, and HSP70, the DAMPs receptor TLR4, and miR328a-3p compared to CAD and controls. Plasma levels of the mir328a-3p target the protective soluble form of the DAMPs receptor for advanced glycation end products (sRAGE), and the antioxidant DJ-1 was decreased in both CAE and CAD compared to controls. In an in vitro human umbilical vein endothelial cells model, circulating levels of S100B, HMGB1, HSP70 as well as CAE patient plasma induced inflammatory responses. The differential expression of the DAMPs S100B, HSP70, HMGB1, and their receptors TLR4 and sRAGE in CAE versus CAD makes them attractive novel biomarkers as therapeutic targets and therapeutics.

## 1. Introduction

Coronary artery ectasia (CAE) is a recognized pathological entity of the coronary arteries with an incidence of 0.3–5.3% observed during coronary angiography [1]. CAE is described as a localized or diffuse dilatation of the epicardial coronary artery that is >1.5-times of the vessel diameter of adjacent normal segments [2]. CAE has often been characterized as an aberrant form of coronary atherosclerosis that may result in such adverse coronary events that include vasospasm, acute coronary syndromes, dissection, and thrombosis [3,4]. 

Plausible causes of CAE include both congenital and acquired disorders. One of the major causes of CAE has been attributed to coronary artery disease (CAD) that in many cases has been observed to co-exist with CAE [3,5,6,7,8]. The pathophysiology, specifically the underlying signaling mechanisms of CAE, are poorly understood. 

Inflammation, endothelial dysfunction, platelet activation, slow flow, microvascular dysfunction, and vascular remodeling may all contribute to CAE [1,2]. Available evidence suggests a critical inflammatory component [3,4]. Strong correlations between the inflammatory cytokines’ interleukin-6 (IL-6), IL-4, T helper-2 lymphocyte (Th-2) immune response, plasma soluble adhesion molecules, and CAE have been described [1,9,10,11].

The most frequently affected vessel is the right coronary artery, and angina pectoris represents the major clinical complaint [1,6]. The long-term outcomes of CAE remain to be defined [2].

The data from several long-term follow-up clinical studies have demonstrated that (i) in Northern Europe, patients with CAE compared to controls have increased cardiovascular mortality [6] and (ii) in a large North American cohort, the angiographic extent of CAE and sluggish coronary flow predict future acute coronary events [7]. Interestingly, the findings of both studies were independent of cardiovascular risk factors and CAD history.

Damage-associated molecular patterns (DAMPs) are endogenous danger molecules that are released from damaged or dying cells and activate the innate immune system by interacting with pattern recognition receptors (PRRs) [12]. DAMPs are ligands predominately for the PRRs, toll-like receptor 4 (TLR4), and the receptor for advanced glycation end-products (RAGE) and include such diverse molecules as high-mobility group box 1 (HMGB1), S100A12, S100B, HSP70, and more recently DJ-1 [12,13,14]. Inflammatory agents generate DAMPs signaling to create a pro-inflammatory state. During cardiac stress, e.g., hypertension, ischemic injury, and metabolic syndrome, release of proinflammatory DAMPs HMGB1 [15] and heat shock protein 60 (HSP60) by cardiomyocytes has been described [16]. 

We aimed to investigate the patterns of expression of a diverse group of DAMPS including S100B, S100A12, HSP70, DJ-1 and their receptors RAGE and TLR4 in plasma of patients with CAE, obstructive CAD, and normal coronary arteries. Our results show differential expression of DAMPS with increases in S100B, S100A12, HMGB1, HSP70, TLR4 and decreases in the soluble form of RAGE and the antioxidant DJ-1 in the plasma of patients with CAE compared to CAD and controls.

## 2. Materials and Methods

### 2.1. Study Population

This study was conducted in compliance with the principles of the Declaration of Helsinki (2013), the principles of good clinical practice and in accordance with all applicable regulatory requirements. The local ethical committees of the participating centers granted consent. A written informed consent was obtained from each study participant prior to the initiation of any study procedures.

This is a prospective cross-sectional evaluation of 2100 consecutive patients, over a 2-year period, between 2008 and 2010, who underwent elective diagnostic coronary angiography in a tertiary hospital. Clinical indications for coronary angiography were angina pectoris and/or positive non-invasive test for myocardial ischemia. Based on coronary angiograms analysis, 29 consecutive individuals with CAE and non-obstructive CAD (Group A), 19 consecutive patients with obstructive CAD but without CAE (Group B), and 14 control subjects with normal coronary arteries (Group C) matched with Group A for age and sex were enrolled in this study. 

Exclusion criteria were the following: valvular heart disease (moderate/severe), uncontrolled hypertension, left or right ventricular dysfunction or history of heart failure, previous percutaneous coronary intervention or coronary artery bypass graft surgery, acute coronary syndromes, autoimmune or connective tissue disease (including past medical history of Kawasaki disease), acute or chronic infectious disease, respiratory disorders including chronic obstructive pulmonary disease and asthma, malignancy and chronic kidney or liver disease, use of steroids or anti-inflammatory medications. 

### 2.2. Angiographic Evaluation

The coronary angiographies were performed by means of the Judkins technique in multiple projections. The coronary angiograms were analyzed by two independent and experienced interventional cardiologists, blinded to patient’s clinical status and laboratory results. A definition of CAE was made based on a fusiform dilatation of the vessel’s lumen above 1.5-fold that of an adjacent normal part of the same vessel. If adjacent normal segments could not be identified, the diameter of the largest coronary artery of the patient was considered as the normal reference value. A narrowing of the lumen of the coronary arteries ≥ 70% was defined as obstructive CAD, whereas lesions < 70% were considered as non-obstructive CAD. Finally, the absence of angiographic ectasia and CAD was considered as normal coronary artery.

### 2.3. Laboratory Methods

Blood samples drawn from the antecubital vein of all participants for the measurement of plasma DAMPs (S100B, S100A12, HMGB1, HSP70, DJ-1 and their receptors RAGE and TLR4) occurred in the morning before the coronary angiography to avoid interference with the inflammatory status due to the plausible interventions that might occur during the catheterization process.

All samples were centrifuged at 3200× *g* for 10 min at a temperature of 4 °C, within an hour after collection. The plasma was immediately separated into aliquots to avoid loss of bioactive cytokines, and rapidly stored in −80 °C, until the assay analysis. The plasma concentrations of human sRAGE, DJ-1, S100B, S100A12, HSP70, TLR4, and HMGB1 were quantified using the respective DuoSet ELISA kit together with the DuoSet ELISA Ancillary Reagent Kit 2 according to the manufacturer’s instructions (R&D Systems Inc., Minneapolis, MN, USA). The sensitivity for each kit was as follows: sRAGE (sensitivity—16.14 pg/mL), DJ-1/Park7 (sensitivity—62.5 pg/mL), S100B (sensitivity—50 pg/mL), S100A12 (sensitivity—7.8 pg/mL), and HSP70 (sensitivity—125.0 pg/mL), TLR4 (sensitivity—31.25 pg/mL), and HMGB1 (sensitivity—18.75 pg/mL) (Novus Biologicals, Centennial, CO, USA). 

### 2.4. Cell Culture

Human umbilical vein endothelial cells (HUVECs) (ATCC-CRL-1730) were cultured in F-12K medium (ATCC-100-010) with 10% FBS (ATCC-30-2020) according to the instructions provided by ATCC. 

HUVECs were transfected with 1 mg of luciferase reporter constructs, and either 20 nmoles of either 328a-3p microRNA mimic (Qiagen, Hilden, Germany, cat # 219600-MSY0000752-5 ‘CUGGCCCUCUCUGCCCUUCCGU), control mimic (Qiagen cat # 339173 -YM00479902), inhibitor (Qiagen, cat# 339121 (YI04101608), 5 ‘CUGGCCCUCUCUGCCCUUCCGU) or control inhibitor (Qiagen cat# 339125-YI00199006) using FuGENE 6 reagent according to the manufacturer’s instructions (Roche, Indianapolis, IN, USA). The media was changed after 1–2 h to serum-free media and the cells were harvested 24 h post-transfection. 

Fetal bovine serum (FBS)–starved HUVECs (for 24 h) in 1 mL of medium were treated for 24 h with either 25, 50, or 100 μL of pooled human plasma from normal controls, patients with either CAD or ectasia. In separate experiments, FBS-starved HUVECs were also treated with endotoxin-free (according to the manufacturer) human recombinant HSP70 (0.5 ng/mL, StressMarq Biosciences, Victoria, BC, Australia), S100B (0.1 ng/mL) or HMGB1 (10 ng/mL, R&D Systems, Minneapolis, MN, USA). 

### 2.5. Luciferase Assay

All luciferase reporter constructs were purchased from Switch-Gear Genomics (Menlo Park, CA, USA). The 3′UTR reporter construct of RAGE gene (cat # S803520 comprised the 3′UTR of the human RAGE gene cloned downstream of a constitutive ribosomal protein L10 (RPL10) promoter and renilla luciferase gene. β-actin control plasmid (cat # S717678) with β-actin promoter was used as control for transfection efficiency. A point mutation of the miR-328a-3p targeting sites in the RAGE 3′UTR (ΔRAGE-miR328a-3p) was generated using the QuickChange Multiple Site-directed Mutagenesis kit (Stratagene, La Jolla, CA, USA). Luciferase activity of each sample was measured three times. All results were normalized to cell lysate. The activity of the luciferase reporter gene was detected using the Dual-Luciferase^®^ Reporter Assay system (Promega Corporation, Madison, WI, USA). Each sample was tested in triplicate, and the relative luciferase activity was calculated as the ratio of renilla to firefly luciferase activity, which was normalized against a blank control.

### 2.6. RT-qPCR for mRNA and miR Expression

mRNA and miRNA were isolated from plasma and HUVECs and reverse-transcribed using QuantiTect SYBR Green PCR kit (Qiagen, cat#204143) or miScript II RT kit (Qiagen, cat#218160) for miR cDNA according to manufacturer’s instruction. Real-time quantitative RT-PCR was performed according to the instructions of the manufacturer (Qiagen). In brief, 1 μL of gene-specific 10 μM PCR primer pair stock of gene (human primers for 18S, RAGE, TNF-α, IL-6-Qiagen, proprietary sequences) or miR (human miR328a-3p-Qiagen cat # 339306- YP00204364 and U6-Qiagen cat# 339306- YP02119464, both proprietary sequences) of interest and 1 μL of cDNA (template) underwent a two-step cycling program, 40 cycles, 10 min at 95 °C, 15 s at 95 °C, and 1 min at 60 °C. In separate experiments, the threshold cycle (C_t_) value for the housekeeping genes 18S (RAGE) and U6 (miR328a-3p) and for the gene of interest in each sample was determined. For each gene of interest, the mean fold-change is shown relative to the gene of interest in healthy controls or vehicle treatment (HUVECs). 

### 2.7. Statistical Analysis

Normality assumptions of continuous variables were examined with Kolmogorov–Smirnov test. Normally distributed variables are expressed as mean and standard deviation values, whereas non-normally distributed variables are presented as median and inter-quartile range values (IQR; 25th–75th percentile). Categorical variables were compared among the CAE, CAD, and control groups using the chi-square test for normally distributed variables and Fischer’s exact test for non-normally distributed variables. One-way analysis of variance (ANOVA) was used to test whether these three groups differed with regards to various continuous parameters of interest. A post hoc analysis with Tukey test was performed for identifying individual levels of statistical significance. The non-parametric Kruskal–Wallis test and post hoc analysis with Mann–Whitney U test were used if the homogeneity of variance assumption was violated. SigmaStat (Systat, San Jose, CA, USA) and GraphPad Prism (Dotmatics, Boston, MA, USA) were used for statistical analysis and generation of graphics, respectively. 

## 3. Results

### 3.1. Baseline Clinical and Laboratory Characteristics 

Baseline characteristics of the three subject groups are presented in Table 1. Across all study groups CAE (Group A), CAD (Group B), and control (Group C), no statistically significant differences were noted with regards to sex, age, hypertension, hyperlipidemia, body mass index (BMI), ejection fraction, systolic and diastolic blood pressure, and medications such as aspirin, angiotensin converting enzyme inhibitors (ACEIs) or angiotensin II receptor blockers (ARBs), statins, nitrates, beta blockers, and calcium channel blockers (CCBs). There was, however, a statistically significant (*p* < 0.05) increase in the number of diabetic patients in the CAD group compared to the CAE and control groups. Also, in the CAE group there was a statistically significant (*p* < 0.05) increase in the number of smokers compared to the CAD and control groups. Univariate logistic regression analysis did not demonstrate any significant relation of the demographic parameters with the incidence of CAE. 

### 3.2. Plasma Levels of sRAGE, S100A12, S100B, HMGB1, TLR4, HSP70 and DJ-1

**Figure 1:**
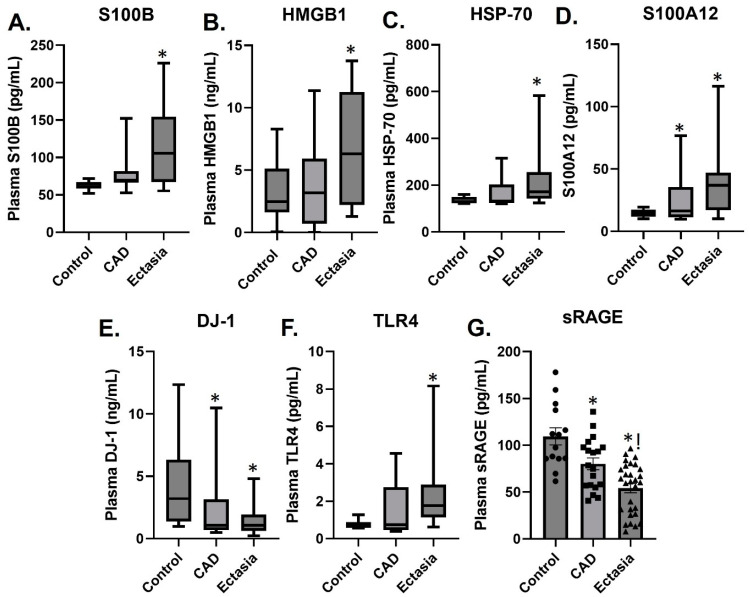
Plasma levels of S100B (pg/mL) (**A**), HMGB1 (ng/mL) (**B**), HSP70 (pg/mL) (**C**), S100A12 (pg/mL) (**D**), DJ-1 (ng/mL) (**E**), TLR4 (pg/mL) (**F**), and sRAGE (pg/mL) (**G**) in healthy controls, patients with obstructive coronary artery disease (CAD) and patients with ectasia and non-obstructive CAD presented as box plots showing median and interquartile range or mean ± SEM (sRAGE). * *p* < 0.05 vs. control, ! *p* < 0.05 vs. CAD. *n* = 14–29.

Plasma levels of the DAMPs S100B, HGMB1, and HSP70 were increased in patients with CAE compared to patients with CAD and controls (Figure 1), whereas the plasma levels of S100A12 were increased, and DJ-1 decreased in both the CAE and CAD groups compared to control (Figure 1). The DAMP receptor TLR4 was increased in patients with CAE, whereas the soluble form of the RAGE receptor sRAGE was decreased in patients with CAD and to a greater extent in CAE patients compared to controls (Figure 1). 

### 3.3. Group Correlations of DAMPs

**Figure 2:**
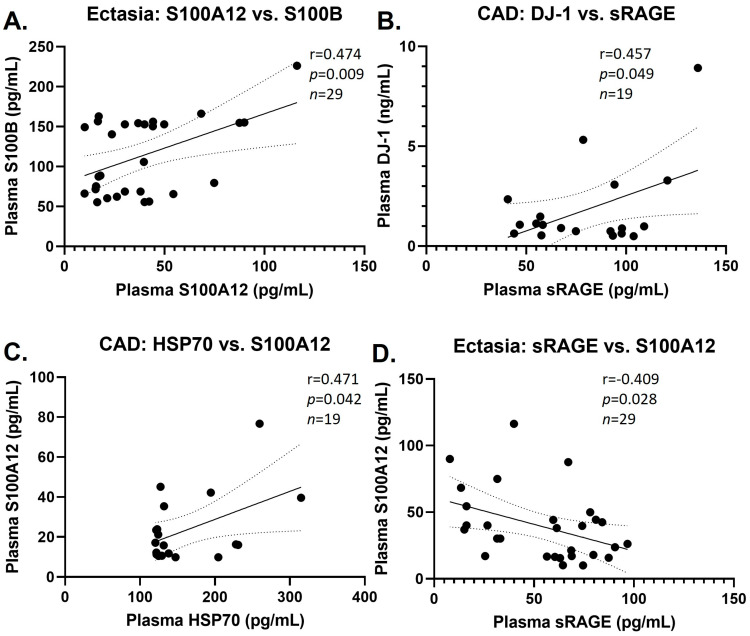
DAMPs correlations. Positive correlations in the CAE group between S100A12 and S100B (**A**), and in the CAD group between DJ-1 and sRAGE (**B**), and between HSP70 and S100A12 (**C**). Negative correlation in the CAE group between sRAGE and S100A12 (**D**).

We tested the correlations between the plasma levels of the selected DAMPs in the various groups. Moderately significant positive correlations were observed in the CAE group between S100A12 and S100B, and in the CAD group between DJ-1 and sRAGE and between SSP70 and S100A12, whereas a moderately significant negative correlation was observed in the CAE group between sRAGE and S100A12 (Figure 2). We did not find any correlations between DAMP plasma levels in CAE, CAD, and controls with baseline characteristics. 

### 3.4. DAMPs Induce Inflammation

**Figure 3:**
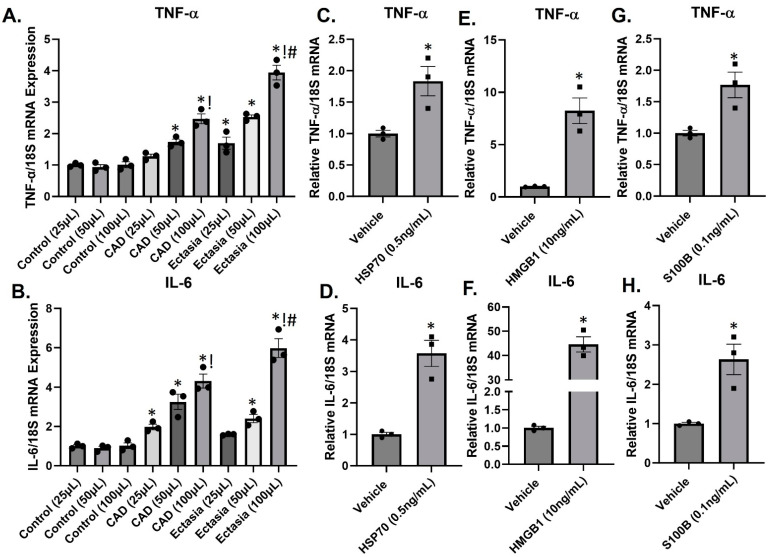
DAMPs and inflammation in HUVECs. Serum-starved HUVECs in 1 mL of medium were treated with pooled plasma (25, 50, or 100 μL) from healthy controls, patients with obstructive coronary artery disease (CAD) and patients with ectasia and non-obstructive CAD for 24 h. Bars are mean of relative TNF-α/18S (**A**), and IL-6/18S (**B**) mRNA expression. * *p* < 0.05 vs. control (25 μL), ! *p* < 0.05 vs. CAD/ectasia (50 μL), # *p* < 0.05 vs. CAD (100 μL). *n* = 3 (each in triplicate). Serum-starved HUVECs were treated with either HSP70 (0.5 ng/mL) (**C**,**D**), HMGB1 (10 ng/mL) (**E**,**F**) or S100B (0.1 ng/mL) (**G**,**H**) for 24 h. Bars are the mean of relative gene/18SmRNA expression. * *p* < 0.05 vs. vehicle. *n* = 3 (each in triplicate).

We have previously shown that CAE is associated with inflammation. Firstly, to test the inflammatory nature of patient plasma, we treated serum-starved HUVECs in 1 mL of medium with pooled plasma at volumes of 25, 50, or 100 μL from patients with CAD, ectasia, or healthy controls. Pooled plasma from CAD and especially ectasia patients induced a dose-dependent increase (1.5–4.5-fold) in TNF-α and IL-6 expression compared to healthy controls (Figure 3A,B). At the highest dose (100 μL), the increase in inflammatory cytokine expression was more pronounced with pooled ectasia plasma compared to CAD. Secondly, to test the inflammatory nature of several circulating DAMPs, we treated serum-starved HUVECs with circulating levels of HSP70, HMGB1, and S100B. Treatment of HUVECs with either HSP70 (0.5 ng/mL, 24 h) HMGB1 (10 ng/mL, 24 h), or S100B (0.1 ng/mL, 24 h) increased the expression of TNF-α and IL-6 approximately 2–45-fold (Figure 3C–H). 

### 3.5. Differential Expression of sRAGE and miR328a-3p in CAE and CAD 

**Figure 4:**
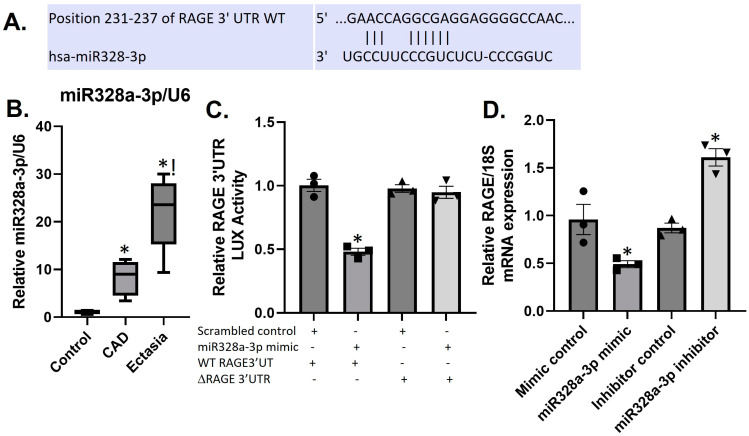
miR328a-3p targets the RAGE3′UTR in HUVECS. Binding site between miR-328a-3p and the 3′-UTR of RAGE mRNA as derived from https://www.targetscan.org/cgi-bin/targetscan/vert_80/view_gene.cgi?rs=ENST00000375065.5&taxid=9606&members=&showcnc=0&shownc=0&showncf1=&showncf2=&subset=1 accessed on 20 January 2023 (**A**). Plasma miR328a-3p levels in controls, patients with obstructive coronary artery disease (CAD) or ectasia patients with non-obstructive CAD presented as box plots showing median and interquartile range (**B**). * *p* < 0.05 vs. controls; ! *p* < 0.05 vs. CAD. *n* = 14–29. HUVECs were transfected with the RAGE–3′UTR luciferase (LUX) reporter in combination with a wild type (WT) miR328a-3p mimic, or ΔmiR328a-3p (target sequence deleted) or scrambled control mimic. Bars are mean + SEM of LUX in cell lysates measured at 24 h post transfection and reported as relative light units normalized to untreated cells (**C**). HUVECs were transfected with either miR328a-3p mimic, miR328a-3p inhibitor, and respective scrambled controls. Bars are mean ± SEM of relative RAGE mRNA (**D**). * *p* < 0.05 vs. scrambles control. *n* = 3 (each in triplicate).

The repression of the RAGE protein in the plasma of CAD and CAE patients suggests involvement of post-transcriptional mechanisms, possibly, involving miRs. Using Targetscan (at https://www.targetscan.org/cgi-bin/targetscan/vert_80/view_gene.cgi?rs=ENST00000375065.5&taxid=9606&members=&showcnc=0&shownc=0&showncf1=&showncf2=&subset=1, accessed on 20 January 2023) we conducted a database search for micro RNAs that would target the human RAGE 3′UTR. One of the miRs that registered a “hit” for RAGE–3′UTR was miR328a-3p. Alignment of the human RAGE-3′UTR with miR328a-3p shows binding of miR328a-3p to position 231–237 (CCU_CCCGUC) of the RAGE 3′UTR (Figure 4A). Indeed, miR328a-3p expression in the plasma of CAD and to a greater extent in CAE patients is increased compared to controls (Figure 4B). 

### 3.6. MiR328a-3p Targets the RAGE-3′UTR in HUVECS

To verify the potential targeting of RAGE by miR328a-3p, we co-transfected a miR328a-3p mimic together with the RAGE–3′UTR reporter into HUVECs. The miR328a-3p mimic reduced RAGE reporter gene expression by over 50% after 24 h, whereas transfection with a scrambled control mimic did not change reporter gene expression (Figure 4C). Confirmation of the role of miR328a-3p in the regulation of RAGE was obtained by deleting the putative miR328a-3p target site within the RAGE–3′UTR of the luciferase reporter. This construct (ΔRAGE-328a-3p), in all other elements identical to the wild-type (WT) RAGE–3′UTR reporter, showed no reduction in reporter gene expression when transfected into HUVECs subjected to miR328a-3p mimic (Figure 4C).

To assess the function of miR328a-3p on endogenously produced RAGE, we transfected HUVECs with miR328a-3p mimic or a specific antimir to miR328a-3p. While transfection of the mimic decreased RAGE, antimir increased RAGE mRNA, whereas the appropriate scrambled controls had no effect on RAGE mRNA (Figure 4D).

## 4. Discussion

The present study sought to identify possible inflammatory pathways implicating DAMPs in the pathophysiology of CAE. We investigated the patterns of expression of the DAMPs S100B, HMGB-1, HSP70, DJ-1 and their receptors RAGE and TLR4 in patients with CAE, obstructive CAD, and normal coronary arteries and the prognostic role of DAMPS in the pathogenesis of CAE. We selected a particular set of DAMPs based on their known physiological properties and our experience from previous research [17]. 

Various aetiologic factors have been associated with CAE formation; however, the exact pathophysiologic mechanism(s) remain(s) to be elucidated. A fundamental aspect of CAE pathology involves the destruction of the vascular media with elastin degeneration and functional loss of the musculoelastic components of the coronary artery media [4,18].

The pivotal role of inflammation in the pathophysiology of CAE has been highlighted by several investigators [1,7,8,9,10,19]. A strong correlation has been identified between increased levels of IL-6, IL-4, plasma soluble adhesion molecules, enhanced Th-2 immune response, and CAE [1,9,10,11]. The cytokine IL-6 augments the up-regulation and activation of matrix metalloproteinases (MMPs), which can subsequently lead to the degradation of extracellular matrix (ECM), destruction of the arterial wall, and eventually in the dilatation of the aorta [20]. Indeed, studies have shown increased levels of serum IL-6 in patients with CAE compared to patients with normal coronaries [1,21]. In this regard, our data show that the treatment of HUVECs with varying volumes of pooled plasma from CAD and ectasia patients induced dose-dependent increases in TNF-α and IL-6.

In the present study, an increased expression of DAMPs, including HMGB-1, S100B, S100A12, HSP-70, and the PRr TLR4 is associated with CAE. Furthermore, treatment of HUVECs with circulating concentrations of the DAMPs S100B, HMGB1, HSP-70, or CAE plasma increased the expression of pro-inflammatory cytokines (TNF-α, IL-6). As such, DAMPs create a pro-inflammatory state which in turn promotes DAMPs generation. 

Advanced glycation end products (AGEs) and their cellular receptors (Receptor of Advanced Glycation End Products, RAGEs) have been associated with long-standing inflammation by increasing cytokines, NF-kB, reactive oxygen species, and matrix metalloproteinases [22,23]. A soluble (s) form of RAGE (sRAGE) is also present in the circulation and consists of shed membrane-bound RAGE ectodomain and secreted endogenous RAGE (esRAGE) [23]. It has been proposed that sRAGE functions as a decoy molecule to bind excess RAGE ligand (e.g., S100 proteins, AGEs, HMGB-1) and, consequently, serves a protective anti-inflammatory role [23]. The levels of sRAGE are decreased, whereas AGEs/RAGEs are increased in aortic tissue and serum of aortic aneurysm patients and correlate positively with the levels of cytokines and metalloproteinases [23]. Additionally, in patients with Kawasaki disease and CAE, the levels of sRAGE are decreased compared to healthy individuals [24]. Our data clearly show that plasma sRAGE is reduced in patients with CAD and to a greater extent in CAE patients compared to healthy controls, resulting in increased DAMP and pro-inflammatory cytokine expression, leading to CAD and/or CAE formation. 

MicroRNAs (miRs) are small, non-coding RNAs of approximately 19–24 nucleotides long that are found in nearly all plants and animals [25]. MiRs function in regulating gene expression for critical cellular processes such as differentiation, cell development expansion, survival, and function [26]. Utilizing the online software https://www.targetscan.org/cgi-bin/targetscan/vert_80/view_gene.cgi?rs=ENST00000375065.5&taxid=9606&members=&showcnc=0&shownc=0&showncf1=&showncf2=&subset=1 accessed on 20 January 2023, we identified miR328a-3p as targeting RAGE. Consequently, we showed that in HUVECS, miR328a-3p directly targets the RAGE 3′UTR, resulting in gene repression. Pathophysiologic relevance may be derived from the observation that sRAGE levels are downregulated, while miR328a-3p levels are increased in CAE patient plasma. Several studies have shown increased plasma levels of miR-328a-3p in acute myocardial infarction (AMI) patients compared to healthy controls [27,28]. Additionally, increased miR328a-3p levels positively correlated with increased risk of heart failure or mortality within 6 months [27]. Increased levels of miR-328a-3p have also been reported in patients with coronary artery aneurysmal disease compared to healthy controls [29].

DJ-1 is a highly conserved, ubiquitously expressed protein that is involved in such biological processes as transcriptional regulation, mitochondrial function, proteolysis, autophagy, and chaperone activity [30,31]. Importantly, DJ-1 functions as an antioxidant by quenching ROS production [30,31]. Following cardiac and vascular surgical procedures, the contribution of DJ-1 in the protection from ischemia reperfusion injury and its antiapoptotic and anti-inflammatory effects has been documented [32]. In patients with aortic aneurysm compared to patients with normal aortic diameter, the thoracic aortic wall DJ-1 expression is decreased [32]. Our findings are consistent with these reports, since in our cohort, both patients with CAE and CAD had decreased plasma levels of DJ-1 compared to controls. It is possible that the attenuated antiapoptotic/anti-inflammatory DJ-1 effects of low plasma DJ-1 may contribute to the formation of CAE. In contrast to the protective effects of DJ-1, a recent study suggests that DJ-1 may also function as a DAMP, as extracellularly released DJ-1 from necrotic neurons after an ischemic stroke elicits sterile inflammation that promotes neuronal injury and neurological deficits [33]. The functional role of DJ-1 as a DAMP in CAE remains to be determined.

HMGB1 is a highly conserved non-histone DNA-binding protein [34]. HMGB1 promotes the secretion of inflammatory cytokines (such as TNF-α, IL-6, IL-1) which in turn activate(s) HMGB1 expression, forming a positive feedback loop, leading to the continuous promotion of inflammation [34]. The binding of HMGB1 to the high-affinity receptor RAGE activates such signaling pathways, as NF-κB, MAPKp38 lead to pro- inflammatory cytokine expression [34]. Increased plasma levels of HMGB1 have been shown in the acute phase of Kawasaki disease [35,36]. Additionally, compared to controls, patients with an abdominal aortic aneurysm (AAA) demonstrated increased aortic tissue HMGB1. This macrophage-produced HMGB1 acted as a trigger for CD4+ T cell-produced IL-17 during AAA formation [37]. Our findings coincide with the above-mentioned data, showing increased plasma levels of HMGB1 in patients with CAE compared to patients with CAD and controls. Importantly, we show circulating levels of HMGB1 observed in CAE patients induced pro-inflammatory responses in HUVECs supporting a direct role of HMGB1 in inflammation.

Among the S100 family of binding proteins S100A8, S100A9, and S100A12 have been identified as DAMPs and linked with cardiovascular disease (CVD) [15,38,39,40]. Clinical data show positive correlations between S100A12 and the severity of coronary and carotid atherosclerosis [40]. We show increased plasma levels of S100A12 in both CAD and CAE patients compared to controls. S100B, predominantly expressed in the nervous system, has been associated with cardiovascular injury following myocardial infarction or norepinephrine stimulation [41]. We have previously shown that S100B released by damaged myocytes post myocardial infarction binds to RAGE, and induces myocyte apoptosis [42]. In our cohort, we show increased S100B plasma levels in patients with CAE compared to CAD and controls. Importantly, we show that circulating levels of S100B in CAE patients induce pro-inflammatory responses in HUVECs and identify extracellular S100B as a DAMP. 

HSPs are chaperone proteins involved in the cellular adaptation to stress, and intracellularly, HSPs mediate protein folding, assembly, transport, and degradation [43]. Several studies have shown that increased serum levels of HSP70 are associated with a low risk of CAD [44]. Importantly, extracellular HSP70 can also act DAMP via Toll-like receptors TLR2 and TLR4 and stimulate immune and inflammatory responses leading to sterile inflammation and propagation of existing inflammation [12,45]. We showed elevated plasma levels of HSP70 in CAE patients compared to CAD and controls. Importantly, treatment of HUVECs with circulating CAE levels of HSP70 induced a marked induction of the inflammatory cytokines TNF-α and IL-6. Elevated plasma levels of HSP70 may contribute to inflammation, but its precise role in CAE is unknown. 

The main limitation of our study is the small sample size, and the findings should be confirmed in a larger cohort. Additionally, we intentionally chose samples from a peripheral vein before any invasive procedure rather than the coronary sinus to minimize the inflammatory status of each patient. However, since CAE and CAD represent a complex pattern of chronic systematic inflammation, it seems unlikely that blood sampling from a peripheral vein would impact the accuracy of our results.

## 5. Conclusions

In conclusion, our findings elucidate the differential expression patterns of the DAMPs S100B, S100A12, HMGB-1, HSP70, the antioxidant DJ-1, the PRs TLR4 and the decoy RAGE receptor sRAGE in CAE. Beyond their pathophysiologic significance, these DAMPs and their receptors could constitute novel diagnostic and therapeutic targets in patients with CAE. 

## Figures and Tables

**Table 1:** Baseline characteristics of the study cohort. Group A: patients with coronary artery ectasia and non-obstructive coronary artery disease; Group B: patients with obstructive coronary artery disease; Group C: patients with normal coronary arteries. BMI: body mass index; LVEF: left ventricular ejection fraction; IQR: interquartile range; SD: standard deviation; SBP: systolic blood pressure; DBP: diastolic blood pressure; ACEIs: angiotensin converting enzyme inhibitors; ARBs: angiotensin receptor blockers; CCBs: calcium channel blockers. * *p* < 0.05. Clinical data were not available for all patients.

General Demographics *	Group A (*n* = 29)	Group B (*n* = 19)	Group C (*n* = 14)	*p* Value
Age ± SD (years)	62.35 ± 10.34	66.42 ± 9.06	61.07 ± 10.19	0.25
Gender (males/females)	23/6	14/5	6/8	0.06
BMI ± SD (kg/m^2^)	29.14 ± 2.66	30.28 ± 3.84	28.01 ± 3.93	0.24
Hypertension (%)	23 (79.3)	17 (89.5)	8 (61.5)	0.16
Hyperlipidemia (%)	22 (75.9)	14 (73.68)	7 (53.8)	0.33
Diabetes (%)	8 (28.6)	11 (57.9)	2 (28.6)	0.03 *
Smoking (%)	18 (62.1)	5 (26.3)	5 (38.5)	0.04 *
LVEF (%), median (IQR)	65 (60.0–70.0)	61.0 (60.0–65.0)	65.0 (60.0–70.0)	0.42
SBP ± SD (mmHg)	129.77 ± 15.69	129.14 ± 18.11	130.00 ± 13.69	0.99
DBP ± SD (mmHg)	70.91 ± 11.41	73.50 ± 12.09	77.22 ± 12.78	0.41
Medications				
Aspirin (%)	20 (71.4)	10 (52.6)	9 (75.0)	0.32
ACEIs (%)	15 (46.4)	8 (42.1)	6 (50.0)	0.91
ARBs (%)	7 (25.0)	6 (31.6)	3 (0.25)	0.87
Statins (%)	18 (64.3)	10 (52.6)	3 (25.0)	0.07
CCBs (%)	5 (17.9)	7 (36.8)	5 (41.7)	0.20
Beta blockers (%)	15 (53.6)	11 (57.9)	4 (33.3)	0.28
Nitrates (%)	8 (28.6)	3 (15.8)	4 (33.3)	0.48

## Data Availability

All data are presented in the manuscript. Any additional details will be made available upon request.
